# BodiMojo: an internet-based body image promotion program

**DOI:** 10.1186/2050-2974-1-S1-O32

**Published:** 2013-11-14

**Authors:** Debra Franko, Rachel Rodgers, Tara Cousineau

**Affiliations:** 1 Northeastern University, USA; 2 Cambridge Innovation Center, USA

## 

BodiMojo, an Internet program designed to promote positive body image in adolescents, was tested with 178 high school students (mean age 15.2 years, 67.6% ethnic minority) in 3 public high schools in the U.S. The BodiMojo group used the program for 4 class periods, while controls participated in their usual health curriculum. Body image measures were given at baseline, post-intervention, and 3-months. Girls reported decreased body dissatisfaction (p < .05), decreased physical appearance comparison (p < .05), and increased appearance satisfaction (p < .05), relative to controls. No effects were found for boys. Moderator analyses revealed a significant Group x Time x Overweight status interaction for body dissatisfaction among girls, with overweight girls reporting greater decreases in body dissatisfaction, p = .012, partial η2 =.15. Among girls, there was a significant Group x Time x Ethnic minority status interaction for the Body Esteem Scale (Appearance subscale), p = .004, partial η2 =.14, and body dissatisfaction, p = .029, partial η2 =.10 with ethnic minority girls reporting greater increases in body appearance esteem than Caucasian girls. BodiMojo appears to be effective in decreasing body image concerns and appearance comparisons among adolescent girls and may be particular effective with some groups.

This abstract was presented in the **Prevention** stream of the 2013 ANZAED Conference.

